# Lobetyolin protects mice against LPS-induced sepsis by downregulating the production of inflammatory cytokines in macrophage

**DOI:** 10.3389/fphar.2024.1405163

**Published:** 2024-05-10

**Authors:** Zhonghua Chen, Yixin Su, Jingtong Ding, Jia He, Lihua Lai, Yinjing Song

**Affiliations:** ^1^ Department of Emergency, Sir Run Run Shaw Hospital, School of Medicine, Zhejiang University, Hangzhou, China; ^2^ Department of Pharmacology, Zhejiang University School of Medicine, Hangzhou, China; ^3^ Teaching Experimental Center of Public Health, Zhejiang University, Hangzhou, China; ^4^ Centre of Biomedical Research, Sir Run Run Shaw Hospital, School of Medicine, Zhejiang University, Hangzhou, China

**Keywords:** Lobetyolin, sepsis, inflammatory cytokines, macrophages, RNA-seq analysis

## Abstract

**Introduction:** Sepsis is a clinical syndrome characterized by dysregulation of the host immune response due to infection, resulting in life-threatening organ damage. Despite active promotion and implementation of early preventative measures and bundle treatments, sepsis continues to exhibit high morbidity and mortality rates with no optimal pharmacological intervention available. Lobetyolin (LBT), the crucial component of polyacetylenes found in *Codonopsis pilosula*, has been scientifically proven to possess potent antioxidant and antitumor properties. However, its therapeutic potential for sepsis remains unknown.

**Methods:** The mice received pretreatment with intraperitoneal injections of LBT, followed by injection with lipopolysaccharide (LPS) to induce sepsis. Peripheral blood samples were collected to detect TNF-α, IL-1β, and IL-6 levels. The survival status of different groups was recorded at various time intervals. RNA-Seq was utilized for the analysis of gene expression in peritoneal macrophages treated with LBT or LPS.

**Results:** In this study, we observed a significant increase in the survival rate of mice pretreated with LBT in LPS induced sepsis mouse model. LBT demonstrated a remarkable reduction in the production of IL-6, TNF-α, and IL-1β in the serum, along with mitigated lung and liver tissue damage characterized by reduced inflammatory cell infiltration. Additionally, through RNA-seq analysis coupled with GO and KEGG analysis, it was revealed that LBT effectively suppressed genes associated with bacterium presence, cellular response to lipopolysaccharide stimulation, as well as cytokine-cytokine receptor interaction involving *Cxcl10, Tgtp1, Gbp5, Tnf, Il1b* and *IRF7* specifically within macrophages. We also confirmed that LBT significantly downregulates the expression of IL-6, TNF-α, and IL-1β in macrophage activation induced by LPS.

**Discussion:** Therefore, our findings demonstrated that LBT effectively inhibits the production of inflammatory cytokines (IL-6, TNF-α, and IL-1β) and mitigates sepsis induced by LPS through modulating macrophages' ability to generate these cytokines. These results suggest that LBT holds promise as a potential therapeutic agent for sepsis treatment.

## 1 Introduction

Sepsis is a life-threatening condition characterized by systemic inflammation resulting from the body’s immune response to an infection (Yang et al., 2023). If left untreated, it can lead to multiple organ dysfunction and ultimately death (Zampieri et al., 2023). Common symptoms of sepsis include fever, tachycardia, tachypnea, and altered mental status (Zheng et al., 2023). The management typically involves administration of appropriate antibiotics along with comprehensive supportive care within a hospital setting (Adegbite et al., 2023). Early recognition and prompt initiation of treatment are crucial for achieving favorable outcomes in septic patients (De Silva et al., 2023). However, despite efforts to improve morbidity and mortality rates associated with sepsis, it remains the leading cause of death among critically ill patients in clinical practice (Borouchaki et al., 2022; Minogue et al., 2023). Additionally, research continues to focus on developing effective strategies for treating sepsis as well.

Macrophages play a crucial role in the innate immune response by recognizing and engulfing pathogens (Smith and Maizels, 2011; Zhang et al., 2023). They detect pathogen-associated molecular patterns through pattern recognition receptors, including toll-like receptors (TLRs), nucleotide-binding and oligomerization domain (NOD) -like receptors, and retinoic acid-inducible gene-I-like receptors (Bird, 2019). Moreover, macrophages secrete inflammatory cytokines such as interleukin (IL) −1, IL-2, IL-6, IFN-gamma, and tumor necrosis factor alpha (TNF-α) to combat infections (McGonagle et al., 2021). In the context of sepsis, macrophages can become hyperactivated, leading to an excessive release of inflammatory molecules that contribute to further tissue damage (Roewe et al., 2020). Ongoing research on macrophage activation in sepsis aims to provide novel insights into the disease’s pathogenesis and identify potential therapeutic targets.

Lobetyolin (LBT), a bioactive compound, is derived from *Codonopsis pilosula* (*C. pilosula*) (Dangshen in Chinese), a perennial herb in the Campanulaceae family (Feng et al., 2017). It has been extensively utilized for centuries in East and Southeast Asian countries as both medicine and food to replenish Qi, nourish blood, enhance immunity, improve appetite, and delay aging (Bailly, 2021). Research indicates that Lobetyolin exhibits diverse biological activities and pharmacological effects including cardioprotective, anti-inflammatory, antioxidant, and antitumor properties (Chen Y. et al., 2021; Tadege et al., 2022; Ni et al., 2023). However, its potential impact on regulating macrophage activation and sepsis remains unknown.

In this study, we have discovered novel insights into the role of LBT in inflammatory diseases. Specifically, our findings demonstrate that LBT exerts a significant inhibitory effect on lipopolysaccharide (LPS)-induced sepsis by suppressing the expression of key pro-inflammatory cytokines such as IL-6, TNF-α, and IL-1β. Moreover, we observed that LBT effectively modulates genes associated with bacterial response, lipopolysaccharide-mediated cellular reactions, and cytokine-cytokine receptor interactions in macrophages. Consequently, our results highlight the remarkable ability of LBT to downregulate the production of IL-6, TNF-α, and IL-1β while alleviating LPS-induced sepsis through its impact on macrophage-mediated inflammatory cytokine production. These findings suggest that LBT holds promising potential as a therapeutic agent for sepsis treatment.

## 2 Materials and methods

### 2.1 Chemicals and reagents

LBT (HY-N0327) was acquired from MedChemExpress Co., Ltd. (Shanghai, China). RPMI-1640 medium, penicillin, streptomycin, and fetal bovine serum were procured from Gibco-BRL (Thermo Fisher Scientific, Inc., Waltham, MA, United States). LPS (tlrl-3pelps; *Escherichia coli* 0111: B4), peptidoglycan (PGN) (tlrl-pgns2; *Bacillus subtilis*), cytosine-phosphate-guanine oligodeoxynucleotide (CpG ODNs) (tlrl-1585), and polyinosinic-polycytidylic acid sodium [Poly (I:C)] (tlrl-picw) were obtained from InvivoGen (CA. United States). Enzyme-linked immunosorbent assay (ELISA) kits for mouse IL-1β (88-7013A-76), IL-6 (88-7064-22), and TNF-α (88-7324-88) were sourced from Invitrogen Life Technologies (Shanghai, China). TRIzol reagent was supplied by Invitrogen Life Technologies (Shanghai, China). SYBR Green PCR Master Mix was provided by Takara, while the ReverTra Ace qPCR RT Kit came from Toyobo (Tokyo, Japan).

### 2.2 Cell culture and treatments

RAW264.7 macrophages (American Type Culture Collection, TIB-71) were cultured in DMEM supplemented with 10% FBS, while Thioglycolate (BD, 3190383, Merck, 1.08191.0500)-elicited mouse peritoneal macrophages were maintained in RPMI-1640 medium containing 10% (vol/vol) FCS. Both cell types were incubated at a temperature of 37°C with a CO2 concentration of 5%. Prior to stimulation with LPS (100 ng/mL), PGN (10 μg/mL), CpG ODNs (10 μM), or Poly (I:C) (10 μg/mL) for either 3 or 6 h, the cells were pre-treated with LTB at a concentration of 10 μM for a duration of 24 h as previously reported (Liu et al., 2022; Ni et al., 2022).

### 2.3 Animal experiments

Male C57BL/6 mice (8 weeks old, *n* = 142) with a mean weight of 26.1 ± 1.2 were obtained from the Model Animal Research Center of Nanjing University. The mice were housed in a 12-h light-dark cycle, with lights turning on at 8:00 a.m., at a constant room temperature of 23°C ± 1°C and humidity maintained at 60% ± 5%. Mice are commonly intraperitoneally anesthetized with a solution of 191.25 mg/kg ketamine and 4.25 mg/kg xylazine. Prior to lipopolysaccharide (LPS) injection with 5 mg/kg, the mice received intraperitoneal injections of LBT 10 mM or phosphate-buffered saline (PBS) for a duration of 24 h as previously mentioned (He et al., 2020; Shao et al., 2020). The mice in the sepsis model group gradually exhibited a series of sepsis symptoms, including piloerection, fever, chills, lethargy, reduced mobility, decreased resistance to stressors, partial eyelid closure, increased secretions, and respiratory distress (Griego et al., 2022). Subsequently, peripheral blood samples, lung tissues, and liver tissues were collected after intraperitoneal administration of LPS at a dose of 5 mg/kg for a period of 4 h to induce sepsis. Carbon dioxide (CO_2_) inhalation was utilized for euthanizing mice. Peripheral blood samples were collected for detection of TNF-α, IL-1β, and IL-6 levels. The survival status of different groups was recorded at various time intervals.

### 2.4 Real-time quantitative PCR (RT-qPCR)

Total mRNA was isolated from cells using an RNA extraction kit (Easy-Do Biotech, Zhejiang, China). Subsequently, 1 μg of total mRNA per sample was utilized for cDNA synthesis employing a ReverTra Ace qPCR RT Kit (Toyobo, Tokyo, Japan) in accordance with the manufacturer’s experimental specifications. Quantitative real-time PCR (qRT-PCR) analysis was performed utilizing a Light Cycler 480 II real-time PCR system (Roche, Basel, Switzerland). The reaction buffer comprised 10 µL of SYBR Green PCR Master Mix from Takara, 7.2 µL of water, 0.4 µL each of forward and reverse primers, and 2 µL of cDNA. The PCR protocol involved an initial denaturation step of 3 min at 94°C, followed by 40 cycles of amplification. Each cycle comprised a denaturation phase of 30 s at 95°C, an annealing phase of 30 s at 58°C, and an extension phase of 1 min at 72°C. Glyceraldehyde phosphate dehydrogenase (GAPDH) was chosen as the reference gene for normalization using the previously described method known as the 2^−ΔΔt^ approach. The primer sequences for candidate genes in quantitative real-time PCR (qRT–PCR) are provided in the results section.

### 2.5 Library construction and sequencing

Library construction and sequencing were performed following previously established protocols (Tang et al., 2023), with minor adjustments. Briefly, three replicate wells of cells were utilized for library construction and sequencing. The RNA integrity was assessed using an Agilent Bioanalyzer 2100 (Santa Clara, CA). For library construction, 1 μg of total RNA per sample was employed according to Illumina’s standard procedure (protocol no. 15008136). To minimize the sequencing of ribosomal RNAs, a poly-(A)-containing mRNA selection procedure was initially conducted. The quality of the libraries was evaluated using the Experion DNA 1K chip method in accordance with the standard protocol. Following cDNA generation, end repair, A-tailing, adaptor ligation, and PCR amplification steps were performed before sequencing the cDNAs on the Illumina HiSeq 2500 platform.

### 2.6 Gene ontology (GO) and KEGG (Kyoto encyclopedia of genes and genomes) enrichment analysis

GO functional significance enrichment analysis was initially performed by mapping all differentially expressed genes (DEGs) to each term in the GO database (http://www.geneontology.org/). Subsequently, the number of genes associated with each term was computed, followed by conducting a hypergeometric test to identify significantly enriched GO items compared to the entire genomic background. The resulting histogram and scatterplot from the GO enrichment analysis depict the distribution of DEGs across enriched GO terms pertaining to biological processes, cell components, and molecular functions.

KEGG pathway enrichment analysis, available at the KEGG database (http://www.genome.jp/kegg/), enables the identification of crucial biochemical metabolic pathways and signal transduction pathways in which DEGs are involved. The extensive pathway information provided by KEGG facilitates a comprehensive understanding of gene functions at a systematic level, encompassing metabolic pathways, genetic information transmission, as well as intricate cellular processes.

### 2.7 ELISA assay

The levels of IL-1β, IL-6, and TNF-α in the blood of mice or cell supernatant were quantified using ELISA. The ELISA experiment was conducted following the manufacturer’s provided protocols (Shao et al., 2020). Briefly described is that 50 μL of serum was added to the plate coated with the capture antibody and incubated for 2 h at room temperature. Unbound antigen was washed away using TBST, followed by addition of HRP-conjugated detection antibody and further incubation for 1 h at room temperature. Excess antibodies were then washed away using TBST. Subsequently, 50 μL of TMB, a chromogenic substrate, was added to the reaction solution and incubated at room temperature for 15 min. The reaction was terminated by adding 25 μL of sulfuric acid solution, and the absorbance was measured at 450 nm.

### 2.8 Histological analysis

The lung and liver tissues from the mice were immersed in 4% paraformaldehyde for 48 h to ensure proper tissue fixation. Subsequently, the fixed skin tissues underwent a dehydration process using a series of alcohol solutions with increasing concentrations ranging from 75% to 100%. Following this, the skin tissues were embedded in wax and sliced into sections that were approximately 5 μL thick. After being blanched with hot water and affixed onto glass slides, the sections were dried within a controlled environment at a temperature of 45°C. Prior to staining, it is essential to remove the wax from the sections using xylene, followed by sequential immersion in high-to-low concentration alcohol solutions before finally rinsing with distilled water. After being dehydrated in 70% and 90% alcohol for a duration of 10 min each, the slices were subsequently stained with an alcohol-eosin staining solution for a period of approximately 2–3 min. Following this, the stained sections underwent dehydration using pure alcohol and were then rendered transparent by xylene prior to microscopic examination.

### 2.9 ALT (alanine aminotransferase), and AST (aspartate aminotransferase) assay

ALT and AST levels of serum following the manufacturer’s instructions (C009-2-1, C010-2-1, Jiancheng, Nanjing China), respectively (Chen S. N. et al., 2021). The reagents and consumables required for the test were prepared in advance following the kit instructions. The test kit was taken out from the 4°C refrigerator and allowed to reach room temperature. Plasma samples from each group were thawed on ice, diluted with primary water at a ratio of 1:10 (5 μL plasma +45 μL pure water), and thoroughly mixed using a suspension instrument. Sample addition was completed as per the instruction sheet. The termination solution was prepared by mixing concentrated termination solution with distilled water at a ratio of 1:9 according to actual requirements. After adding the termination solution, the well plate was gently shaken horizontally to ensure thorough mixing. Following a 15-min incubation period at room temperature, the absorbance at 510 nm was measured using a microplate reader to determine the OD of each well, and corresponding viability values were calculated based on the standard curve.

### 2.10 Statistical analysis

Results are presented as the mean ± standard error of the mean (SEM). Statistical analysis was performed using GraphPad Prism 6.0 software. Significance between two groups was assessed using a two-tailed Student’s *t*-test. For the mouse survival study, data were analyzed utilizing the Log rank test (Mantel-Cox). The significance level was set at *p < 0.05.

## 3 Results

### 3.1 Pre-treatment with LBT conferred protection against sepsis induced by LPS in mice

**FIGURE 1:**
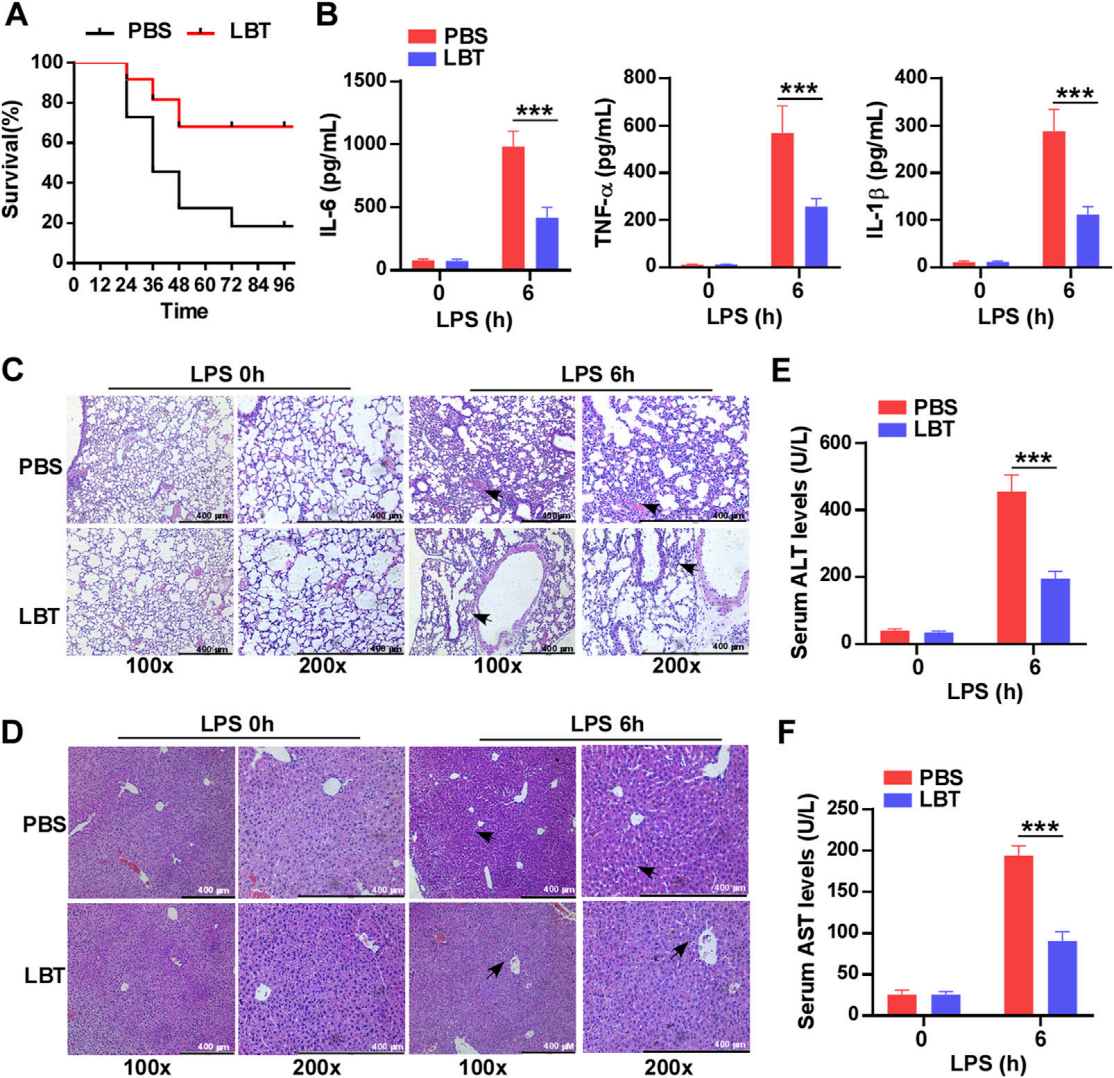
Pre-treatment with LBT protected mice against LPS-induced sepsis. **(A)** C57BL/6 mice (*n* = 10 per group) were intraperitoneally administered LBT (10 mM) or PBS for 24 h, followed by injection of LPS (5 mg/kg). Mouse survival was assessed and the data were analyzed using the Log rank test (Mantel-Cox). Statistical significance was indicated as *p < 0.05. **(B)** ELISA analysis was performed to measure IL-6 **(B)**, TNF-α **(C)**, and IL-1β **(D)** levels in the serum from C57BL/6 mice (*n* = 6 per group) pre-treated with LBT (10 mM) or PBS for 24 h, followed by injection of LPS (5 mg/kg) for 6 h. **(C,D)** H&E staining analysis was conducted on lung **(E)** and liver samples **(F)** from C57BL/6 mice by above treatment. scale bar = 400 μm. The arrows indicated infiltrating inflammatory cells **(E,F)**. Serum levels of ALT (G) and AST (H) were measured at 6 h after intraperitoneal injection of LPS in C57BL/6 mice by above treatment. Data are shown as the mean ± s.e.m. and are representative of three independent experiments. Data are shown as the mean ± s.e.m. and are representative of three independent experiments. The *p* values were determined using a two-tailed Student’s *t*-test. Statistical significance was denoted as **p < 0.01 and ***p < 0.001.

To investigate the role of LBT in sepsis, mice were pretreated with LBT for 24 h prior to LPS administration. The survival rate was recorded over a period of 4 days and demonstrated that administration of LBT significantly enhanced survival compared to the control group (Figure 1A). Furthermore, our findings revealed that LBT effectively reduced the production of IL-6, TNF-α, and IL-1β in the serum of mice following LPS injection (Figure 1B). Histological examination using H&E staining exhibited less severe lung and liver tissue damage along with reduced inflammatory cell infiltration in the pre-treated mice administered with LBT after intraperitoneal injection of LPS (Figures 1C, D). To further validate the hepatoprotective effects of LBT, we also assessed the serum levels of ALT and AST, and found the elevated levels of ALT and AST were significantly inhibited by LBT treatment (Figures 1E, F). Our results indicated that LBT could protect against sepsis induced by LPS in mice.

### 3.2 LBT regulated the gene expression of peritoneal macrophages

**FIGURE 2:**
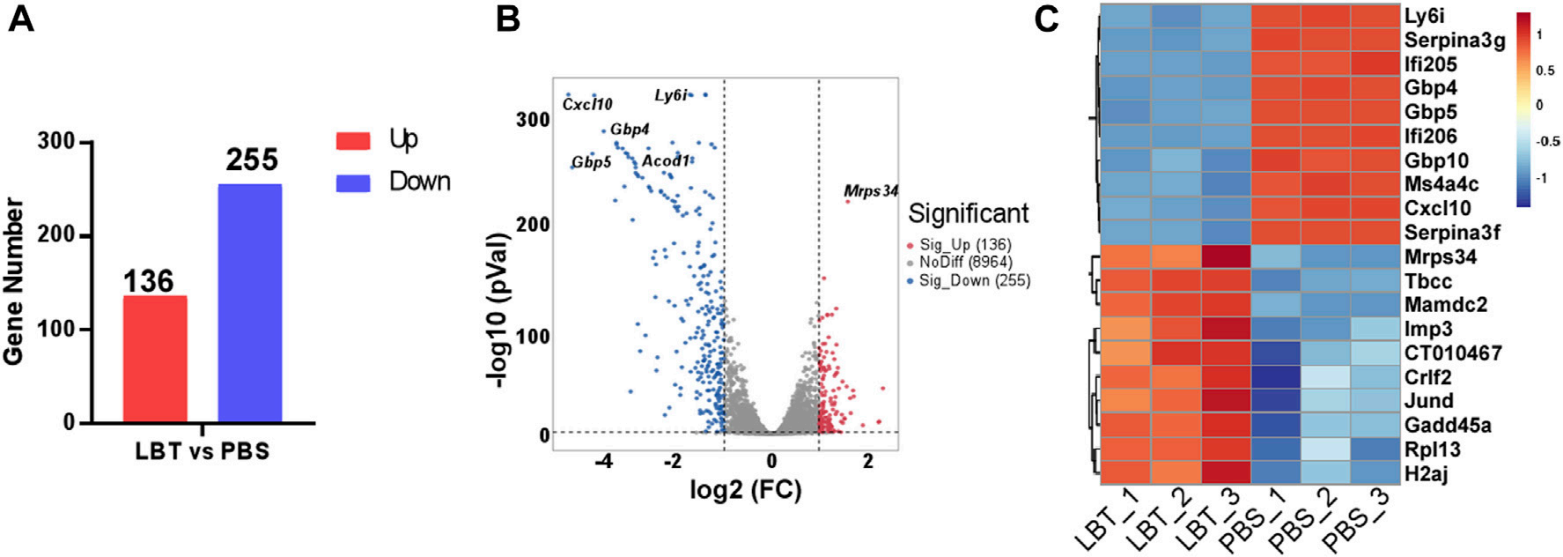
LBT regulated the gene expression of peritoneal macrophages. Peritoneal macrophages were treated with LBT (10 μM) or PBS for a duration of 24 h. The mRNAs were extracted and subsequently subjected to RNA sequencing. **(A)** DEGs between the control group and the 10 μM LBT treatment group are shown. **(B)** Volcano plots showed all genes from the data obtained by RNA sequencing after treatment of peritoneal macrophages with 10 μM LBT for 0 or 24 h. **(C)** Venn diagram showing genes that were differentially regulated in both the 10 μM LBT-treated cell groups.

Macrophages play a pivotal role in the progression of sepsis by releasing excessive amounts of pro-inflammatory cytokines, which leads to systemic inflammation and tissue damage. Therefore, we investigated whether LBT could modulate the gene expression of macrophages. Peritoneal macrophages were treated with 10 μM LBT, and their transcriptional gene expression profiles were analyzed using RNA sequencing. Following LBT treatment, we observed 391 DEGs in macrophages: 136 genes were significantly upregulated while 255 genes showed dramatic downregulation (Figures 2A, B). Figure 2C illustrated the top 20 most significantly upregulated genes and top 20 most dramatically downregulated genes upon LBT treatment. LBT markedly enhanced the expression of mitochondrial ribosomal protein S34 (*Mrps34*), ribosomal protein L13 (*Rpl13*), junD proto-oncogene (*Jund*), cytokine receptor like factor 2 (*Crlf2*), and IMP U3 small nucleolar ribonucleoprotein 3 (*Imp3*) among others, while significantly suppressing the expression of C-X-C motif chemokine ligand 10 (*Cxcl10*), lymphocyte antigen 6 family member I (*Ly6i*), guanylate-binding protein 10 (*Gbp10*), and guanylate-binding protein 10 (*Gbp4*) et al. (Figure 2C). These findings underscored the significant regulatory impact of LBT on macrophage gene expression profiles.

### 3.3 GO and KEGG analysis of DEGs regulated by LBT in peritoneal macrophage

**FIGURE 3:**
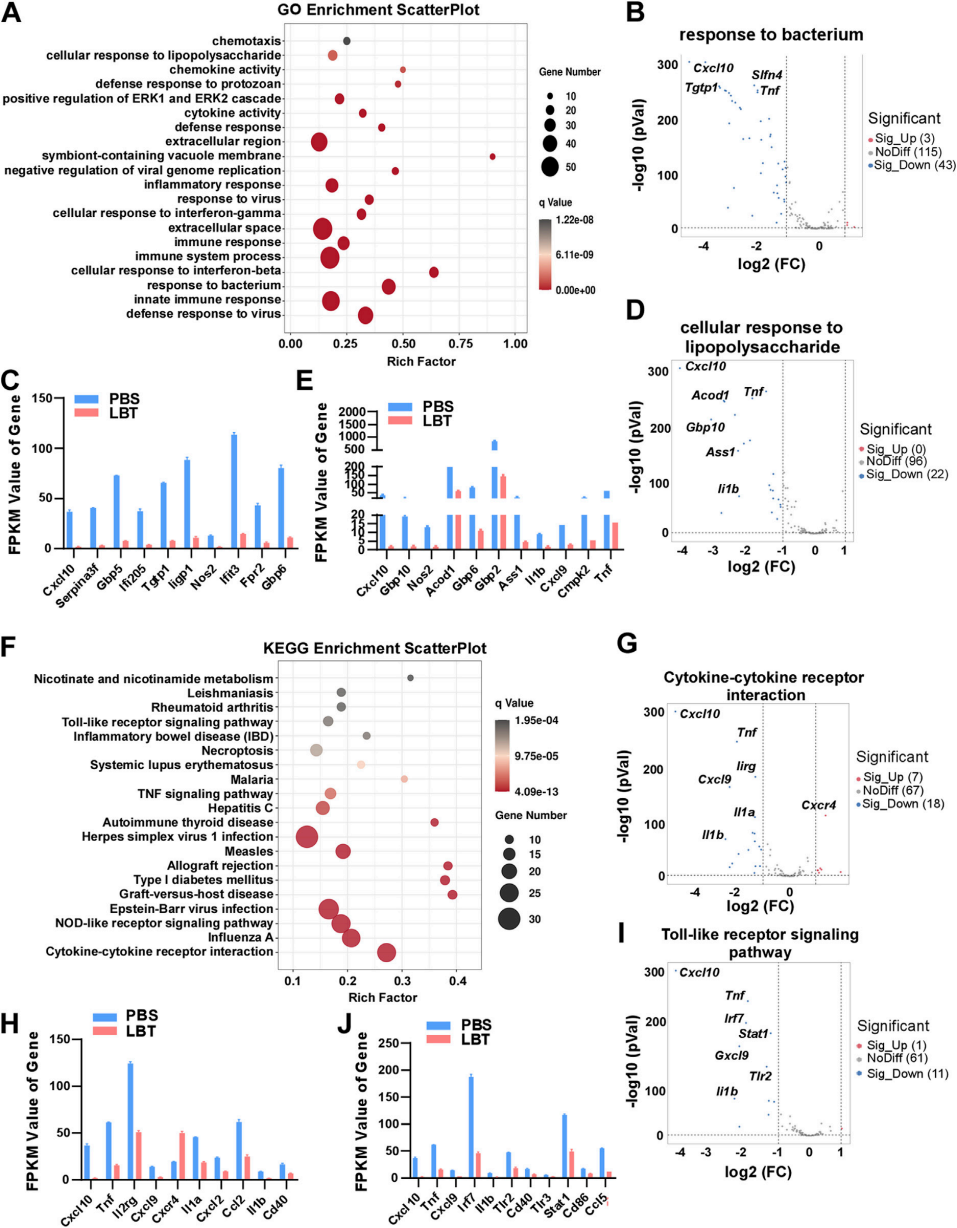
GO and KEGG analysis of DEG regulated by LBT in peritoneal macrophage. **(A)** Genes clustered into the four most significantly changed GO terms are shown. **(B,C)** Volcano plots showed response to bacterium related genes **(B)**, and Top 10 downregulated DEGs with FPKM values > 0 **(C)** in peritoneal macrophages after LBT treatment. **(D,E)** Volcano plots showed cellular response to lipopolysaccharide related genes **(D)**, and Top 10 downregulated DEGs with FPKM values > 0 **(E)** in peritoneal macrophages after LBT treatment. ***, *q* < 0.001 **(F)** Scatter plot shows the top 20 most significant KEGG pathways of peritoneal macrophages treated with or without 10 μM LBT for 24 h. The dot size indicates the number of DEGs enriched in the corresponding KEGG pathway. Dot colors indicate p values. **(G,H)** Volcano plots showed response to cytokine-cytokine receptor interaction **(G)**, and Top 10 downregulated DEGs with FPKM values > 0 **(H)** in peritoneal macrophages after LBT treatment. **(I,J)** Volcano plots showed cellular response to toll-like receptor signaling pathway **(I)**, and Top 10 downregulated DEGs with FPKM values > 0 **(J)** in peritoneal macrophages after LBT treatment. **, *q* < 0.001.

GO functional significance enrichment analysis was performed on all DEGs, revealing the top 20 dysregulated pathways in the GO database (Figure 3A). Surprisingly, LBT exhibited significant regulation of genes associated with response to bacterium, cellular response to lipopolysaccharide, innate immune response, and defense response to virus in peritoneal macrophages (Figure 3A). Notably, LBT significantly suppressed the expression of 43 genes involved in bacterium response, including *Cxcl10*, T cell specific GTPase 1 (*Tgtp1*), guanylate-binding protein 5 (*Gbp5*) and *Tnf* (Figures 3B, C). In addition, supplementation of LBT exhibited a remarkable downregulation in the expression of 22 genes associated with cellular response to lipopolysaccharide, including *Cxcl10*, aconitate decarboxylase 1 (*Acod1*), *Il1b*, and *Tnf* expression (Figures 3D, E). These findings indicated that LBT significantly attenuated the differential gene expressions related to bacterium response and cellular response to lipopolysaccharide as annotated in GO.

Then, we conducted KEGG pathway enrichment analysis on the DEGs affected by LBT in macrophages. Figure 3F illustrates the top 20 dysregulated KEGG pathways in peritoneal macrophages following LBT treatment. Notably, cytokine-cytokine receptor interaction, toll-like receptor signaling pathway, NOD-like receptor signaling pathway, and Influenza A were identified as the most significantly dysregulated signaling pathways (Figure 3F). Specifically, LTB downregulated the expression of eight genes associated with cytokine-cytokine receptor interaction including *Cxcl10*, *Tnf*, *Il1b* and *Il1a*; while upregulating seven genes such as C-X-C motif chemokine receptor 4 (*Cxcr4*) in peritoneal macrophages (Figures 3G, H). In addition, supplementation of LBT significantly suppressed the expression of 11 genes related to the toll-like receptor signaling pathway, including *Tnf*, *Il1b* and interferon regulatory factor 7 (I*rf7*) (Figures 3I, J). These findings indicated that LTB effectively downregulated cytokine-cytokine receptor interaction and toll-like receptor signaling pathway-associated genes in peritoneal macrophages.

### 3.4 RNA-seq analysis of peritoneal macrophage treated with LBT and LPS

**FIGURE 4:**
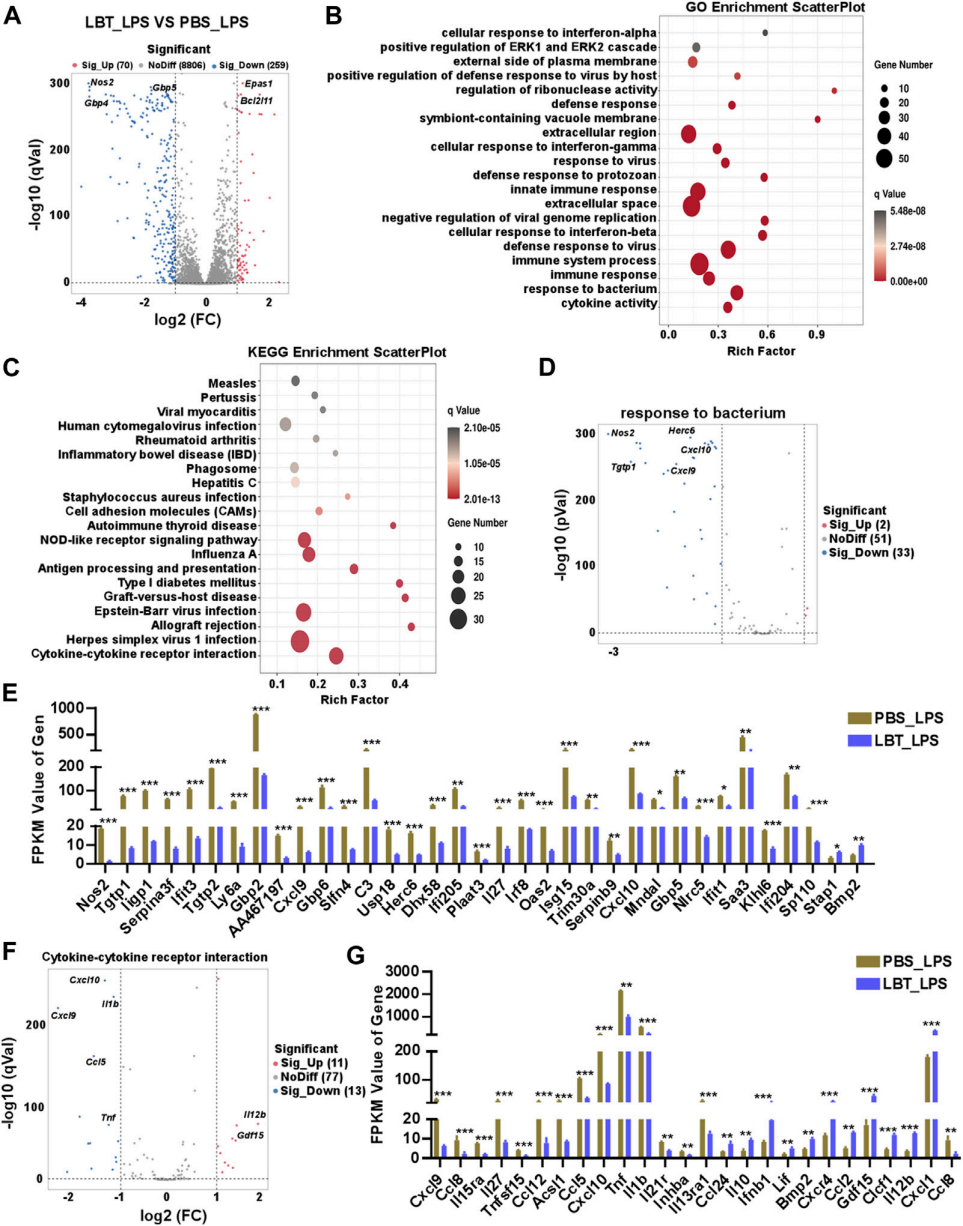
RNA-seq analysis of peritoneal macrophage treated with LBT and LPS. **(A)** Volcano plots showed all genes from the data obtained by RNA sequencing in 10 μM LBT pre-treated peritoneal macrophages after 100 ng/mL LPS stimulation. **(B)** Scatter plot shows the top 20 most significant GO of macrophages with LBT and LPS treatment. **(C)** Scatter plot shows the top 20 most significant KEGG pathways of macrophages with LBT and LPS treatment. **(D,E)** Volcano plots showed response to bacterium related genes **(D)**, and DEGs with FPKM values > 0 **(E)** in peritoneal macrophages after LBT and LPS treatment. **(F,G)** Volcano plots showed response to cytokine-cytokine receptor interaction **(B)**, and DEGs with FPKM values > 0 **(C)** in peritoneal macrophages after LBT treatment. *q < 0.05, **q < 0.01, *q* < 0.001.

Next, we investigated the impact of LBT on peritoneal macrophages following LPS stimulation using RNA-seq analysis. Our findings revealed a total of 329 DEGs in peritoneal macrophages after treatment with both LBT and LPS. Among these DEGs, 70 genes exhibited significant upregulation while 259 genes displayed substantial downregulation (Figure 4A). Supplementary Figures 1A, B showcased the top ten most significantly upregulated and dramatically downregulated genes upon LBT treatment. GO analysis revealed significant alterations in cytokine activity, response to bacterium, and immune response-associated genes in LBT-treated peritoneal macrophages upon LPS stimulation (Figure 4B). The KEGG signaling pathway analysis of the DEGs demonstrated that the pathways primarily affected were cytokine-cytokine receptor interaction-associated genes, herpes simplex virus infection, and allograft rejection (Figure 4C). Additionally, we observed downregulation of 33 genes associated with response to bacterium (Figure 4D), including nitric oxide synthase 2 (*Nos2*), *Tgtp1,* and *Cxcl9*. In contrast, only two genes exhibited significant upregulation (Figure 4E; Supplementary Figure 1C). The genes involved in cytokine-cytokine receptor interaction (Figure 4F), such as *Tnf*, C-C motif chemokine ligand 5 (*Ccl5*), and *Il1b* were significantly downregulated in LBT-treated peritoneal macrophages following LPS stimulation (Figure 4G; Supplementary Figure 1D). These findings indicated that LTB can effectively modulate the cytokine-cytokine receptor interaction and the expression of bacterium-associated genes in macrophages during LPS treatment.

### 3.5 LBT inhibited the expression of TNF-α and IL-6 in macrophages

**FIGURE 5:**
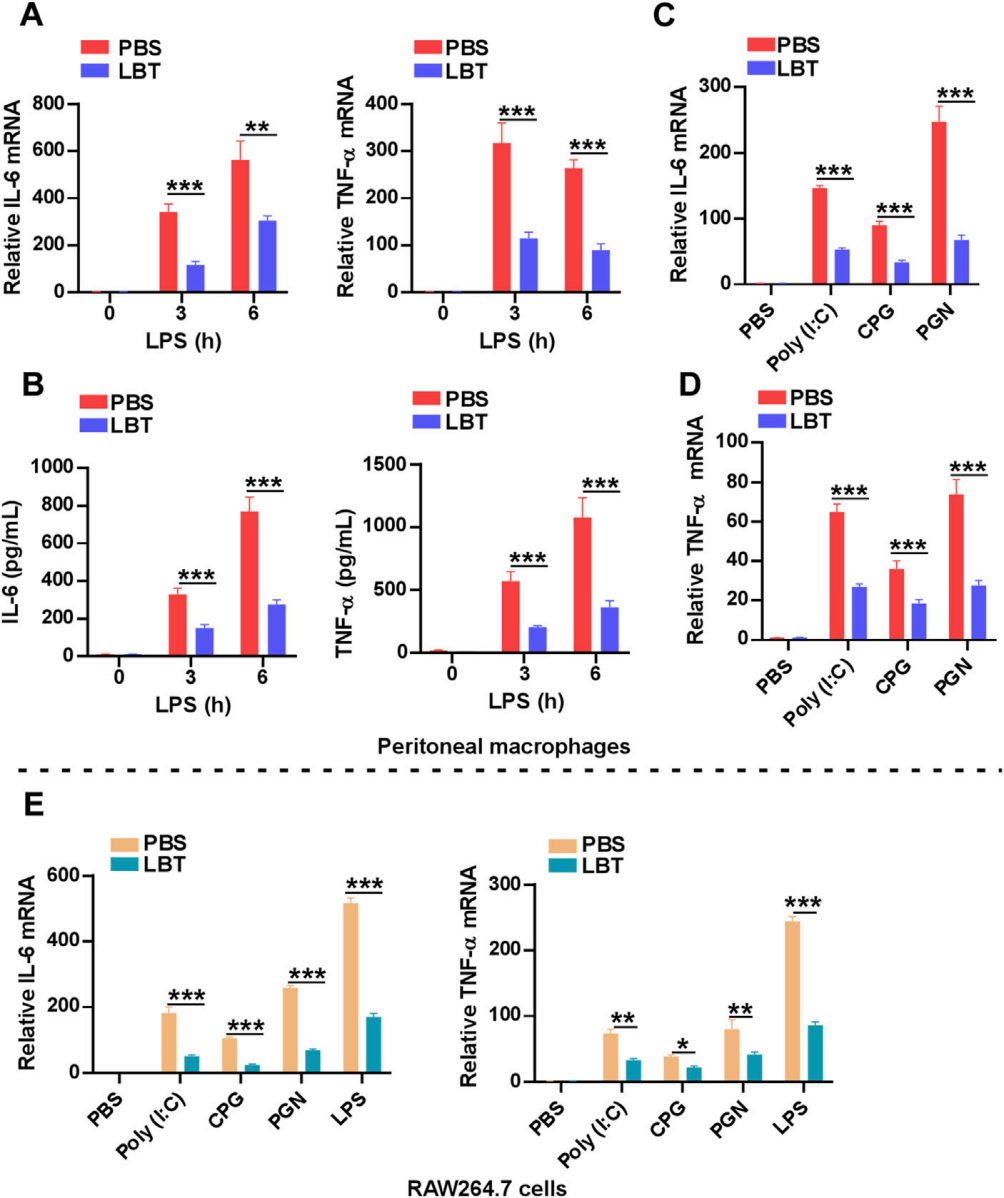
LBT inhibited the expression of TNF-α and IL-6 in macrophages. **(A,B)** RT-PCR **(A)** and ELISA **(B)** analysis of IL-6 and TNF-α in peritoneal macrophages after pretreatment with LBT for 24 h followed with LPS stimulation for indicated times. **(C,D)** RT-PCR analysis of IL-6 **(C)** and TNF-α **(D)** in peritoneal macrophages after pretreatment with LBT for 24 h followed with poly (I:C), CPG, and PGN stimulation for 3 h. **(E)** RT-PCR analysis of IL-6 and TNF-α in RAW264.7 cells after pretreatment with LBT for 24 h followed with LPS, poly (I:C), CPG, and PGN stimulation for 3 h. Data are shown as the mean ± s.e.m. and are representative of three independent experiments. Three replicate wells were included in each group. **, *q* < 0.01, ***, q < 0.001.

To further validate the downregulation of cytokine-cytokine receptor interaction related genes by LBT, we employed RT-PCR and ELISA techniques to assess the production of TNF-α and IL-6. Following LPS stimulation, both mRNA and protein levels of TNF-α and IL-6 exhibited a significant decrease in peritoneal macrophages upon treatment with LBT (Figures 5A, B). Furthermore, upon activation of macrophages with TLR3 ligands [Poly (I:C)], TLR9 ligands (CPG), or TLR2 ligands (PGN), a significant decrease in mRNA expression levels of TNF-α and IL-6 was observed in peritoneal macrophages following treatment with LBT (Figures 5C, D), as well as in RAW264.7 cells (Figure 5E). These findings unequivocally demonstrate that LTB effectively suppresses the expression of TNF-α and IL-6 in macrophages.

## 4 Discussion

Sepsis, a life-threatening condition resulting from an imbalanced host response to infection, has surpassed myocardial infarctions in terms of mortality rate and emerged as a prominent cause of death among non-cardiac patients in intensive care units (Nazer et al., 2023; Yang et al., 2023). In China, herbal medicines are frequently employed alongside conventional sepsis management strategies (Usmani et al., 2021). For instance, XueBiJing is an intravenous formulation derived from Chinese herbs that has been developed for the management of sepsis and multiple organ dysfunction syndrome (Cheng et al., 2024). It has shown potential in reducing the 28-day mortality rate among critically ill patients by modulating the septic response (Song et al., 2019). Additionally, *C. pilosula*, a traditional Chinese medicinal herb with a history spanning thousands of years, has demonstrated its ability to attenuate the progression of sepsis (Zheng et al., 2014). However, further research is needed to elucidate the active pharmaceutical ingredient and molecular mechanisms involved in these therapeutic effects. In this study, we observed that LBT, a bioactive compound derived from *C. pilosula*, exhibited significant efficacy in enhancing the survival rate of mice following LPS injection. Additionally, our findings demonstrated that LBT effectively downregulated the levels of IL-6, TNF-α, and IL-1β in serum samples while inhibiting inflammatory cell infiltration within lung and liver tissues induced by LPS-induced sepsis. These results strongly suggest that LBT possesses potential therapeutic properties for attenuating sepsis triggered by LPS administration. However, our study only utilized the LPS-induced sepsis model, and there are some differences with sepsis in the clinic. Therefore, we will use peritoneal contamination and infection or cecal ligation and puncture to induce sepsis in mice, and even conduct clinical trials to further explore whether LTB can effectively inhibit the occurrence of sepsis. Lobetyolin protects mice against LPS-induced sepsis by downregulating the production of inflammatory cytokines in macrophage.

LBT has been scientifically proven to possess cardioprotective, anti-inflammatory, antioxidant, and antitumor properties (Ni et al., 2022). Research suggests that LBT effectively mitigates muscle injury induced by chronic kidney disease through the inhibition of ferroptosis via activation of the Hedgehog signaling pathway (Ni et al., 2023). Moreover, in gastric cancer and breast cells, LBT demonstrates remarkable anticancer effects by suppressing cell proliferation and inducing cell apoptosis through the downregulation of ASCT2 (He et al., 2020; Chen Y. et al., 2021). Also, LBT has been discovered to exhibit potent inhibitory effects on xanthine oxidase both *in vitro* and *in vivo* (Yoon and Cho, 2021). This enzyme plays a crucial role in the breakdown of purines and their subsequent conversion into uric acid (Liu et al., 2022). Moreover, LBT demonstrates remarkable cardioprotective properties along with anti-arrhythmic activity (Liu et al., 2018). Additionally, it effectively stimulates angiogenesis, leading to vascular morphological abnormalities while exhibiting low toxicity (Li et al., 2015). Furthermore, LBT exhibits the ability to safeguard BV2 microglial cells against damage induced by oxygen-glucose deprivation/reperfusion through its regulation of BV2 phenotypic polarization and reduction of inflammatory responses. This is achieved by suppressing the production of TNF-α, IL-6, inducible nitric oxide synthase (iNOS), and cluster of differentiation CD 206 (Wang et al., 2023). In this study, our findings demonstrated that LBT Lobetyolin effectively protects mice from sepsis induced by LPS by downregulating the production of pro-inflammatory cytokines such as TNF-α, IL-6, and IL-1β in macrophages. Additionally, RNA-Seq, and GO analysis revealed LBT could regulate the cytokine activity, response to bacterium, and immune response-associated genes, including *Nos2*, *Tgtp1,* and *Cxcl9* in peritoneal macrophages upon LPS stimulation. KEGG analysis showed LBT could affect the cytokine-cytokine receptor interaction, herpes simplex virus infection, and allograft rejection related genes such as *Tnf*, *Ccl5*, and *Il1b.* Our results demonstrated that LBT protects mice against LPS-induced sepsis by downregulating the production of inflammatory cytokines in macrophage. However, the role in other immune cells remains unclear. We will continue to explore the affects of LBT on the inflammatory response of other immune cells, such as T cells, dendritic cells, and B cells.

During the hyperinflammatory phase of sepsis, macrophages play a crucial role in monitoring the infection through their ability to phagocytose bacteria, present antigens, and produce significant amounts of cytokines such as TNF-α, IL-6, and IL-1β (Nascimento et al., 2021). Henceforth, our investigation focuses on determining whether LBT can regulate the inflammatory gene expression of macrophages without LPS treatment. The results from GO analysis demonstrate that LBT effectively downregulates the response to bacterium and lipopolysaccharide-associated genes including *Cxcl10*, *Tgtp1*, *Gbp5 Acod1*, *Il1b*, and *Tnf*. KEGG analysis showed LTB significantly downregulated the expression of genes related cytokine-cytokine receptor interaction, including *Tnf*, *Il1b* and *Il1a*, and inhibited toll-like receptor signaling pathway, such as *Tnf*, *Il1b* and *IRF7*. Our findings suggest that LBT may exert an impact on the macrophages’ capacity to produce inflammatory cytokines, potentially through modulation of cellular metabolism or epigenetic regulation. However, the precise mechanism by which LBT affects the ability of macrophages to produce inflammatory cytokines remains unclear. Additionally, we found the epigenetic regulation related gene such as H1.2 linker histone, cluster member (*H1F2*) was significantly upregulated in macrophages after LBT treatment. *H1F2* encodes the H1-2 protein, which has been reported to have the ability to regulate H3K27me3 and H3K36me2, as well as inhibit gene expression. Therefore, we will explore whether LBT regulates macrophage activity through H1F2, thereby inhibiting the inflammatory response.

## 5 Conclusion

In conclusion, our study has uncovered novel insights into the role of LBT in inflammatory diseases. Specifically, we have demonstrated that LBT exerts significant reductions in TNF-α, IL-6, and IL-1β levels while mitigating LPS-induced sepsis through the inhibition of genes associated with bacterial infection, cellular response to lipopolysaccharide, and cytokine-cytokine receptor interaction. Consequently, we propose that LBT holds promising potential as a therapeutic intervention for sepsis.

## Data Availability

The data presented in the study are deposited in the Sequence Read Archive repository, accession number PRJNA1090007.
