# Supplementary material for: Lobetyolin protects mice against LPS-induced sepsis by downregulating the production of inflammatory cytokines in macrophage
Source: Front Pharmacol. 2024 May 10;15:1405163. doi: 10.3389/fphar.2024.1405163 (PMC11116692; doi:10.3389/fphar.2024.1405163)
Supplement: Supplementary file 1 [file DataSheet1.docx]

**Supplementary figure 1. RNA-seq analysis of peritoneal macrophage treated with LBT and LPS.**

**
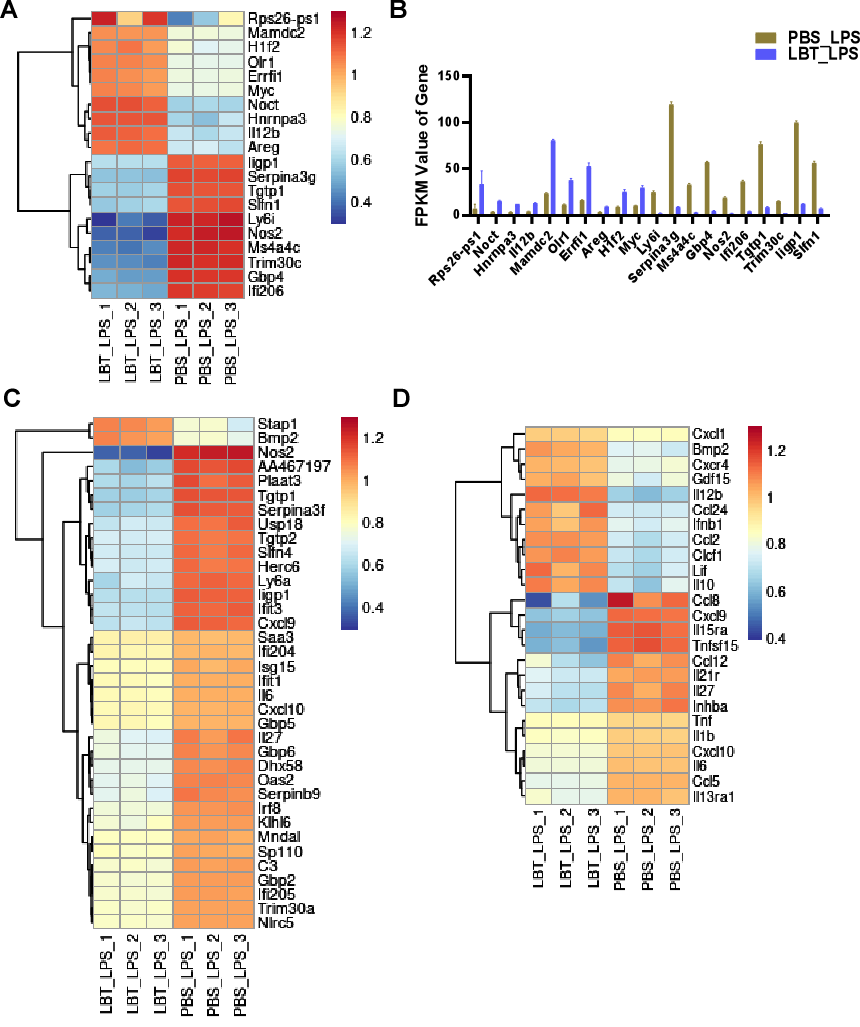
**

A-B. Heat map (A) and FPKM (B) value showed top 20 DEGs in LBT pre-treated peritoneal macrophages after 100 ng/ml LPS stimulation.

C-D. Heat map showed DEGs associated with bacterium related genes (C), and cytokine-cytokine receptor interaction (D) in LBT pre-treated peritoneal macrophages after 100 ng/ml LPS stimulation.
